# The role of the addition of ovarian suppression to tamoxifen in young women with hormone-sensitive breast cancer who remain premenopausal or regain menstruation after chemotherapy (ASTRRA): study protocol for a randomized controlled trial and progress

**DOI:** 10.1186/s12885-016-2354-6

**Published:** 2016-05-19

**Authors:** Hyun-Ah Kim, Sei Hyun Ahn, Seok Jin Nam, Seho Park, Jungsil Ro, Seock-Ah Im, Yong Sik Jung, Jung Han Yoon, Min Hee Hur, Yoon Ji Choi, Soo-Jung Lee, Joon Jeong, Se-Heon Cho, Sung Yong Kim, Min Hyuk Lee, Lee Su Kim, Byung-In Moon, Tae Hyun Kim, Chanheun Park, Sei Joong Kim, Sung Hoo Jung, Heungkyu Park, Geum Hee Gwak, Sun Hee Kang, Jong Gin Kim, Jeryong Kim, Su Yun Choi, Cheol-Wan Lim, Doyil Kim, Youngbum Yoo, Young-Jin Song, Yoon-Jung Kang, Sang Seol Jung, Hyuk Jai Shin, Kwan Ju Lee, Se-Hwan Han, Eun Sook Lee, Wonshik Han, Hee-Jung Kim, Woo Chul Noh

**Affiliations:** Department of Surgery, Korea Cancer Center Hospital, Korea Institute of Radiological and Medical Sciences, Seoul, Republic of Korea; Department of Surgery, University of Ulsan, Asan Medical Center, Seoul, Republic of Korea; Department of Surgery, Samsung Medical Center, Sungkyunkwan University School of medicine, Seoul, Republic of Korea; Department of Surgery, Yonsei University College of Medicine, Seoul, Republic of Korea; Center for Breast Cancer, National Cancer Center, Goyang, Republic of Korea; Department of Internal Medicine, Seoul National University Hospital, Cancer Research Institute, Seoul National University College of Medicine, Seoul, Republic of Korea; Department of Surgery, Ajou University, School of Medicine, Suwon, Republic of Korea; Department of Surgery, Chonnam National University Hwasun Hospital, Gwangju, Republic of Korea; Department of Surgery, Cheil General Hospital and Women’s Healthcare Center, Dankook University College of Medicine, Seoul, Republic of Korea; Department of Internal Medicine, Korea University Anam Hospital, Seoul, Republic of Korea; Department of Surgery, Yeungnam University Hospital, Daegu, Republic of Korea; Department of Surgery, Gangnam Severance Hospital, Yonsei University, Seoul, Republic of Korea; Department of Surgery, Dong-A University Hospital, Busan, Republic of Korea; Department of Surgery, Soonchunhyang University College of Medicine, Cheonan Hospital, Cheonan, Republic of Korea; Department of Surgery, Soonchunhyang University Colleage of Medicine, Seoul, Republic of Korea; Division of Breast & Endocrine Surgery, Hallym University Sacred Heart Hospital, College of Medicine, Hallym University, Anyang, Republic of Korea; Department of Surgery, Mokdong Hospital, Ewha Womans University, Seoul, Republic of Korea; Department of Surgery, Inje University Busan Paik Hospital, Busan, Republic of Korea; Department of Surgery, Sungkyunkwan University School of Medicine, Kangbuk Samsung Hospital, Seoul, Republic of Korea; Department of Surgery, Inha University Hospital, Inha University, Incheon, Republic of Korea; Department of Surgery, Chonbuk National University Medical School, Jeonju, Republic of Korea; Department of Breast Surgery, Gachon University Gil Hospital, Incheon, Republic of Korea; Department of Surgery, Inje University Sanggye Paik Hospital, Inje University College of Medicine, Seoul, Republic of Korea; Department of Surgery, Keimyung University School of Medicine, Daegu, Republic of Korea; Departments of Surgery, Seoul National University Boramae Medical Center, Seoul, Republic of Korea; Department of Surgery, Chungnam National University Hospital, Daejeon, Republic of Korea; Department of Surgery, KangDong sacred heart hospital, Hallym university, Seoul, Republic of Korea; Department of Surgery, Soonchunhyang University College of Medicine, Bucheon Hospital, Bucheon, Republic of Korea; Department of Surgery, Kangseo Mizmedi Hospital, Seoul, Republic of Korea; Department of Surgery, Konkuk University School of Medicine, Seoul, Republic of Korea; Department of Surgery, Chungbuk National University College of Medicine and Medical Research Institute, Cheongju, Republic of Korea; Department of Surgery, Eulji University Hospital, Daejeon, Republic of Korea; Department of Surgery, Seoul St. Mary’s Hospital, Medical College of The Catholic University of Korea, Seoul, Republic of Korea; Breast and thyroid care center, Department of Surgery, Myongji Hospital, Goyang, Republic of Korea; Department of Surgery, Daejeon St. Mary’s Hospital, The Catholic University of Korea, Daejeon, Republic of Korea; Department of Surgery and Cancer Research Institute, Seoul National University College of Medicine, Seoul, Republic of Korea

**Keywords:** Ovarian function suppression, Goserelin, Tamoxifen, Adjuvant endocrine therapy, Premenopause, Breast cancer

## Abstract

**Background:**

Ovarian function suppression (OFS) has been shown to be effective as adjuvant endocrine therapy in premenopausal women with hormone receptor-positive breast cancer. However, it is currently unclear if addition of OFS to standard tamoxifen therapy after completion of adjuvant chemotherapy results in a survival benefit. In 2008, the Korean Breast Cancer Society Study Group initiated the ASTRRA randomized phase III trial to evaluate the efficacy of OFS in addition to standard tamoxifen treatment in hormone receptor-positive breast cancer patients who remain or regain premenopausal status after chemotherapy.

**Methods:**

Premenopausal women with estrogen receptor-positive breast cancer treated with definitive surgery were enrolled after completion of neoadjuvant or adjuvant chemotherapy. Ovarian function was assessed at the time of enrollment and every 6 months for 2 years by follicular-stimulating hormone levels and bleeding history. If ovarian function was confirmed as premenopausal status, the patient was randomized to receive 2 years of goserelin plus 5 years of tamoxifen treatment or 5 years of tamoxifen alone. The primary end point will be the comparison of the 5-year disease-free survival rates between the OFS and tamoxifen alone groups. Patient recruitment was finished on March 2014 with the inclusion of a total of 1483 patients. The interim analysis will be performed at the time of the observation of the 187th event.

**Discussion:**

This study will provide evidence of the benefit of OFS plus tamoxifen compared with tamoxifen only in premenopausal patients with estrogen receptor-positive breast cancer treated with chemotherapy.

**Trial registration:**

ClinicalTrials.gov Identifier NCT00912548. Registered May 31 2009. Korean Breast Cancer Society Study Group Register KBCSG005. Registered October 26 2009.

## Background

Many prospective randomized trials have shown that adjuvant endocrine therapy, such as with tamoxifen or ovarian function suppression (OFS), provides a disease free survival benefit for young patients with hormone receptor-positive breast cancer [1–3]. However, there is insufficient information whether adding OFS to standard tamoxifen treatment for premenopausal patients is an effective therapy in reducing disease recurrence.

Premenopausal breast cancer patients with hormone receptor-positive disease have a worse prognosis than postmenopausal breast cancer patients with hormone receptor-positive disease [4, 5]. This difference in survival may be due to tamoxifen resistance in premenopausal women [5]. Theoretically, the combination of OFS and tamoxifen therapy could overcome tamoxifen resistance in premenopausal women. However, in the absence of clinical evidence of a definitive survival benefit associated with OFS plus standard tamoxifen therapy, additional toxicities from OFS treatment complicate recommendation of this treatment regimen. Therefore, it is important to identify patients most likely to benefit from additional OFS treatment.

The results of the Suppression of Ovarian Function Trial (SOFT), a randomized, phase 3 trial conducted by The International Breast Cancer Study Group (IBCSG), showed no significant benefit from the addition of ovarian suppression to tamoxifen in overall patients [6]. However, in women who remained premenopausal and were at sufficient risk of recurrence to warrant adjuvant chemotherapy, the addition of OFS improved disease outcomes. In SOFT, ovarian function was assessed by serum E2 measurement just one time within 8 months after chemotherapy regardless of menstruation. However, it is assumed that examination at only one time point may be insufficient to evaluate ovarian function after chemotherapy. The patients who regain ovarian function later may lose the chance to benefit from the addition of ovarian suppression treatment. The patients who regain ovarian function later may lose their chance to benefit from the addition of ovarian suppression treatment. As there is no standard method to predict the resumption of ovarian function at the time of chemotherapy completion, we decided to evaluate ovarian function repeatedly for 2 years.

The Korean Breast Cancer Society Study Group has designed and initiated a randomized phase III trial comparing OFS plus tamoxifen versus tamoxifen only after chemotherapy in young women with estrogen receptor-positive breast cancer (ASTRRA); participants include those with premenopausal status or those who have regained ovarian function after the completion of neoadjuvant or adjuvant chemotherapy. The primary objective of this study is to compare the 5-year disease-free survival rates between the two groups.

## Methods/design

### Study design and setting

ASTRRA is a phase III open-label, prospective, randomized, multicenter investigator initiated clinical trial. The trial was designed to evaluate the combination of 2 years of goserelin plus 5 years of tamoxifen (OFS group) versus 5 years of tamoxifen alone (tamoxifen alone group) as adjuvant endocrine therapy according to ovarian function after the completion of neoadjuvant or adjuvant chemotherapy in patients with estrogen receptor-positive breast cancer. The Korean Breast Cancer Society Study Group coordinates the trial, and the Steering Committee oversees the trial. The institutional review board of Korea Cancer Center Hospital was approved the protocol version 1.3 [K-0902-004-009]. The study protocol was approved by each institutional review board of all participating centers as well. Table 1 shows the list of participating centers. All patients provided written informed consent before enrollment.

**Table 1:** List of participating centers of ASTRRA trial

Names of institutes
Ajou University, School of Medicine
Cheil General Hospital and Women’s Healthcare Center, Dankook University College of Medicine
Chonbuk National University Medical School
Chonnam National University Hwasun Hospital
Chungnam National University Hospital
Chungbuk National University College of Medicine and Medical Research Institute
Daejeon St. Mary’s Hospital, The Catholic University of Korea
Dong-A University Hospital
Eulji University Hospital
Gachon University Gil Hospital
Gangnam Severance Hospital, Yonsei University
Hallym University Sacred Heart Hospital, College of Medicine, Hallym University
Inha University Hospital, Inha University
Inje University Busan Paik Hospital
Inje University Sanggye Paik Hospital, Inje University College of Medicine
KangDong sacred heart hospital, Hallym university
Kangseo Mizmedi Hospital
Keimyung University School of Medicine
Konkuk University School of Medicine
Korea Cancer Center Hospital, Korea Institute of Radiological and Medical Sciences
Korea University Anam Hospital
Mokdong Hospital, Ewha Womans University
Myongji Hospital
National Cancer Center
Samsung Medical Center, Sungkyunkwan University School of medicine
Seoul National University Boramae Medical Center
Seoul National University Hospital, Seoul National University College of Medicine
Seoul St. Mary’s Hospital, Medical College of The Catholic University of Korea
Soonchunhyang University College of Medicine, Cheonan Hospital
Soonchunhyang University Colleage of Medicine
Soonchunhyang University College of Medicine, Bucheon Hospital
Sungkyunkwan University School of Medicine, Kangbuk Samsung Hospital
University of Ulsan, Asan Medical Center
Yeungnam University Hospital
Yonsei University College of Medicine

**Table 2:** Demographics of randomized patients

	Tamoxifen only group (B + D group, *N* = 655)	Ovarian function suppression group (C + E group, *N* = 634)	*P*-value
Age(mean, years)	39.7 ± 4.1	39.6 ± 4.1	0.580
Stage			
I	178 (27.2 %)	169 (26.7 %)	0.977
II	335 (51.1 %)	332 (52.4 %)	
III	121 (18.5 %)	113 (17.8 %)	
Unidentified	21 (3.2 %)	20 (3.2 %)	
Lymph node status			
Negative	279 (42.6 %)	275 (43.4 %)	0.927
Positive	371 (56.6 %)	355 (56.0 %)	
Unidentified	5 (0.8 %)	4 (0.6 %)	
Histology			
Invasive ductal carcinoma	573 (87.5 %)	560 (88.3 %)	0.917
Invasive lobular carcinoma	32 (4.9 %)	26 (4.1 %)	
Others	42 (6.4 %)	41 (6.5 %)	
Unidentified	8 (1.2 %)	7 (1.1 %)	
Histologic grade			
G1	95(14.5 %)	118 (18.6 %)	0.229
G2	359 (54.8 %)	323 (50.9 %)	
G3	160 (24.4 %)	151 (23.8 %)	
Unidentified	41(6.3 %)	42 (6.6 %)	
Chemotherapy regimen			
Anthracycline + cyclophosphamide	184 (28.1 %)	185 (29.2 %)	0.782
Anthracycline + cyclophosphamide followed by taxane	324 (49.5 %)	318 (50.2 %)	
Anthracycline + taxane	30 (4.6 %)	29 (4.6 %)	
5-fluorouracil + anthracycline + cyclophosphamide	74 (11.3 %)	73(11.5 %)	
Others	21 (3.2 %)	14(2.2 %)	
Unidentified	22 (3.4 %)	15 (2.4 %)	
Operation			
Total mastectomy	268 (40.9 %)	248 (39.1 %)	0.762
Breast conserving surgery	382 (58.3 %)	382 (60.3 %)	
Unidentified	5 (0.8 %)	4 (0.6 %)	

### Patients

The trial enrolled premenopausal women ≤ 45 years of age with histologically confirmed estrogen receptor-positive, stage I–III, primary invasive breast cancer treated with definitive surgery and chemotherapy. Premenopausal status for inclusion criteria was defined as having a regular menstruation history at the time of diagnosis. Estrogen receptor positivity was determined as expression of estrogen receptor in at least 10 % of tumor cells as determined by immunohistochemistry or 10 fmol/mg cytosol protein as determined by a dextran-coated charcoal ligand binding assay.

Receipt of neoadjuvant or adjuvant chemotherapy was required, and the standard regimens were allowed except CMF. Adjuvant trastuzumab therapy for patients with human epidermal growth factor receptor-2-positive disease was permitted, although it was not considered as chemotherapy.

We excluded patients with other primary malignancies within the last 5 years, except for adequately treated in situ carcinoma of the cervix, basal cell carcinoma, or squamous cell carcinoma of the skin. In addition, patients with thrombocytopenia, those currently treated with anti-coagulant agents, and patients that were pregnant, lactating, or treated with investigational drugs within the previous 4 weeks before baseline assessment were excluded.

### Study design

**Fig. 1:**
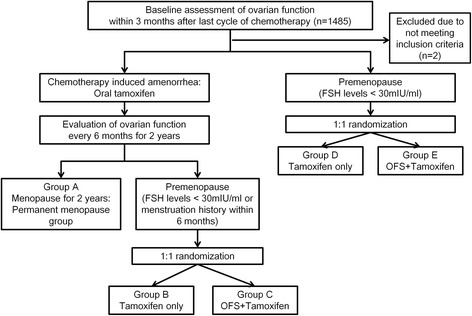
Study design

The first screening test to evaluate ovarian function was performed within 3 months of the final dose of chemotherapy. Premenopausal status at the first screening test was defined by serum follicular stimulating hormone (FSH) levels < 30 mIU/ml. At 6, 12, 18, and 24 months following the baseline assessment, ovarian function status is to be evaluated by menstruation status and serum FSH levels. Regaining premenopausal status is defined by FSH levels < 30mIU/ml or bleeding history within 6 months of each visit. Study visits will be every 6 months for 5 years and at least yearly thereafter, according to each institute’s routine practice. If the patient does not regain satisfy the definition of being premenopausal during the 24 months after enrollment, the patient will be categorized to the permanent menopause group (group A). At each visit, newly confirmed premenopausal patients will be randomly assigned in a 1:1 ratio to the OFS group (group C or group E) or the tamoxifen alone group (group B or group D). The OFS group is treated with 3.6 mg subcutaneous injection goserelin (Zoladex® [D-Ser(But)^6^ Azgly^10^ luteinizing-hormone-releasing hormone]; AstraZeneca) every 28 days for 2 years plus oral tamoxifen at a dose of 20 mg daily for 5 years. The tamoxifen only group is treated with oral tamoxifen at a dose of 20 mg daily for 5 years. Randomization is performed by means of an internet-based system and is stratified according to lymph node status (negative versus positive) and institutes (Fig. 1). Data are collected and stored in a digital case report form.

### Primary and secondary end points

The primary end point is to compare the 5-year disease-free survival rates between the OFS and tamoxifen alone groups, particularly among patients with premenopausal status (assessed every 6 months for 2 years) after the completion of chemotherapy. Disease-free survival is defined as the time from enrollment to the detection of invasive recurrence of breast cancer (local, regional, or distant metastasis), contralateral breast cancer, secondary malignancy, or death without breast cancer recurrence. Patients who are still alive without any event at the time of the analysis will be censored.

Secondary end points are (1) to compare overall survival rates between groups, (2) to compare 5-year disease-free survival rates between postmenopausal patients treated with tamoxifen and premenopausal patients treated with OFS plus tamoxifen, (3) to determine the tolerability of tamoxifen with or without goserelin.

### Sample size calculation and statistics

Planned enrollment was at least 1234 patients. Initially, the design projected that 2 years of accrual, plus 5 years of additional follow-up would be sufficient to observe the target of 374 disease-free survival events across the two treatment arms, with 85 % power to detect 7 % reduction in hazard with OFS plus tamoxifen versus tamoxifen alone. In 2010, because of a slower-than-expected enrollment rate, the steering committee extended the accrual period from 2 years to 4 years.

An intent-to-treatment analysis and per-protocol analysis will be performed. The disease-free survival rate will be evaluated using the Kaplan-Meier method. The log-rank test will be used to compare the treatment groups. Multivariate analyses will be performed using Cox’s proportional hazards model.

### Trial progress

Recruitment was closed on March 2014. Between March 2009 and March 2014, 1485 patients were screened, and 1483 patients from 35 institutes in South Korea were enrolled in this study. On January 12 2015, 634 patients were randomized to the OFS group, and 655 patients were randomized to the tamoxifen only group (Table 2). Eighty patients were classified as permanent menopause status. Another 114 patients continue to exhibit a status of chemotherapy-induced amenorrhea, and the ovarian function of these patients is being evaluated every 6 months. All of the patients received chemotherapy before randomization. Node-positive disease was present in 56.3 % of the patients. The first interim analysis will be performed when 50 % of the planned disease-free survival events (187 events) have occurred.

## Discussion

In South Korea, 48.7 % of newly diagnosed breast cancer patients in 2011 were premenopausal and less than 50 years of age [7]. Although the total number of patients is smaller than that of western countries, the rate of premenopausal patients is higher in South Korea. The Korean Breast Cancer Society has been focused on developing optimal tailored therapy for these patients because of the relatively higher proportion of premenopausal patients in the Korean breast cancer patient population. In 2008, the Korean Breast Cancer Society Study Group initiated the ASTRRA trial to answer the following questions: (1) whether disease free survival benefits could be achieved with the addition of OFS to standard 5-year tamoxifen treatment after the completion of neoadjuvant or adjuvant chemotherapy in premenopausal young women with estrogen receptor-positive disease, and (2) whether delayed OFS treatment could reduce disease recurrence in patients with recovered ovarian function who experienced chemotherapy-induced amenorrhea and who were treated with standard tamoxifen therapy.

Results from phase III trials including OFS, as well as a meta-analysis of these trials, might help to advance current knowledge of the survival advantage gained with addition of OFS treatment [8–14]. Of these trials, SOFT was a randomized, three-arm, phase III trial designed to investigate the role of OFS in women with premenopausal status either after completion of (neo)adjuvant chemotherapy or following surgery alone. The SOFT trial included three arms: (1) tamoxifen only for 5 years, (2) tamoxifen for 5 years + OFS for 5 years, and (3) exemestane for 5 years + OFS for 5 years [15]. One of the comparisons in the SOFT trial was tamoxifen + OFS versus tamoxifen alone, similar to the comparison in the ASTRRA trial. Although the studies have some resemblance, there are significant distinctions between the study design of the SOFT trial and the ASTRRA trial. First, the ASTRRA trial has only included women aged ≤ 45 years. Because standard endocrine therapy takes at least 5 years, older premenopausal women could experience natural, spontaneous menopause during endocrine therapy, and this would obscure the effect of OFS. Second, in contrast to the SOFT trial population, only 53 % of which were treated with chemotherapy, all participants in the ASTRRA trial received neoadjuvant or adjuvant chemotherapy before enrollment. Thus, ASTRRA trial focuses more on the role of OFS after completing chemotherapy. Third, ovarian function was assessed only one time (based on estradiol levels) at the time of randomization in the SOFT trial, within 8 months after completing chemotheapy. However, resumption of ovarian function occurs in about 60 % of women younger than 45 years of age within 2 years after completing chemotherapy [16, 17]. We assume that patients who recently regained ovarian function may lose the chance to benefit from the addition of OFS treatment. Therefore, in the ASTRRA trial, ovarian function will be evaluated by menstruation history or FSH levels every 6 months from the time of enrollment for at least 2 years. Until now, 1286 (86.7 %) patients in the ASTRRA trial are premenopausal or have regained premenopausal status after chemotherapy, and only 80 (5.4 %) patients have been classified to the permanent menopausal group after 2 years of observation. Examination at only one time point may thus be insufficient to evaluate ovarian function after chemotherapy.

The proportion of patients with regained ovarian function is slightly higher in the ASTRRA trial than in other reports. This might be caused by the exclusion of patients treated with CMF regimens [16, 17]. Because most patients treated with CMF do not recover from chemotherapy-induced amenorrhea, we excluded patients who had received the CMF regimen [8, 16, 17]. In contrast to the CMF regimen, modern non-CMF chemotherapy regimens result in less permanent amenorrhea after treatment. The NSABP B-30 trial assessed menstrual status after various non-CMF chemotherapy regimens at baseline and every 6 months over 24 months. The incidence of amenorrhea 12 months after random assignment was 69.8 % for sequential doxorubicin and cyclophosphamide followed by docetaxel, 57.7 % for concurrent docetaxel-doxorubicin-cyclophosphamide, and 37.9 % for concurrent docetaxel-doxorubicin (*P* < 0.001) [18]. Although CMF is an effective chemotherapy regimen for breast cancer patients, use of the CMF regimen in young patients is currently decreasing in South Korea. Thus, we believe that the removal of the CMF regimen from the trial’s acceptable chemotherapy regimen list is compatible with recent trends in the care of young women with breast cancer. Another reason for the high rate of ovarian function resumption in the ASTRRA trial would be the relatively young age of participants. The NSABP B-30 trial showed that age is significantly related to the incidence of chemotherapy-induced amenorrhea [18].

The important advantage of the ASTRRA trial study design is the repeated evaluation of ovarian function. The longitudinal evaluation of ovarian function may help to select the most appropriate patients to receive additional OFS treatment, thereby avoiding unnecessary side effects. OFS causes menopausal symptoms and bone mass loss [19, 20]; menopausal symptoms, such as vasomotor symptoms, vaginal dryness, vaginal discharge, anxiety, depression, or sleep disturbances, significantly affect quality of life [19]. Sometimes these symptoms result in low compliance or destroy the physician-patient relationship. Because there is yet no reliable biomarker to select patients most likely to benefit from OFS, continuous checking of ovarian function may facilitate this patient selection.

Currently, the ASTRRA trial has closed to accrual, with a total 1483 enrolled patients. Through the ASTRRA trial, we can determine optimal endocrine therapy based on real-time ovarian function status for each premenopausal breast cancer patient with estrogen receptor-positive disease who received neoadjuvant or adjuvant chemotherapy.

### Ethics approval and consent to participate

The institutional review board of Korea Cancer Center Hospital was approved the protocol [K-0902-004-009]. The study protocol was approved by each institutional review board of all participating centers as well (Table 1).

### Consent for publications

Not applicable.

### Availability of data and materials

The dataset supporting the conclusions of this article will is not available until the final report of this trial to ovoid bias on the analysis.

## Abbreviations

CMF: cyclophosphamide, methotrexate, and fluorouracil
FSH: follicular stimulating hormone
IBCSG: International Breast Cancer Study Group
OFS: ovarian function suppression
SOFT: suppression of ovarian function trial
